# Impact of visual distractors in virtual reality environments on sustained attention behavioral performance and EEG characteristics

**DOI:** 10.3389/fnhum.2025.1705660

**Published:** 2025-11-28

**Authors:** Xiaowen Ai, Yuhao Wang, Peng Wang, Suogang Wang

**Affiliations:** 1 Neuroengineering Laboratory, School of Biomedical Engineering and Technology, Tianjin Medical University, Tianjin, China; 2 Institute of Biomedical Engineering, Chinese Academy of Medical Sciences and Peking Union Medical College, Tianjin, China

**Keywords:** virtual reality, visual distractors, sustained attention, EEG, P300, entropy

## Abstract

**Introduction:**

This study investigates the effects of visual distractors in virtual reality (VR) environments on sustained attention, focusing on how visual distraction modulates neural mechanisms of attentional allocation and regulation.

**Methods:**

Behavioral and electroencephalographic (EEG) data were collected from 66 participants performing a Go/No-go continuous performance test (CPT) in a virtual classroom under conditions with (Y-D) and without (N-D) visual distractors. We analyzed behavioral performance (commission/omission errors, multipress, reaction time), event-related potential (P300) characteristics (latency, amplitude), and nonlinear dynamics (sample entropy, fuzzy entropy) of the EEG signals.

**Results:**

Behavioral results revealed that visual distractors significantly increased commission errors, omission errors, and multipress (all *p* < 0.001), with no significant difference in reaction time. EEG analysis demonstrated that distractors significantly prolonged P300 latency, particularly at CPz, Pz, and Oz electrodes, and increased P300 amplitude at Fz, FCz, and Oz. Furthermore, both sample entropy and fuzzy entropy values were significantly higher under distraction conditions in the frontal, central, and parietal regions.

**Discussion:**

These findings indicate that visual distractors disrupt cognitive processes related to visual information integration, attentional control, and decision-making, leading to decreased behavioral performance and increased neural complexity. This study deepens the understanding of the neural mechanisms of attention processing under ecological conditions and provides a scientific basis for optimizing educational environments and developing attention assessment tools based on neuroengineering.

## 1 Introduction

Attention, a fundamental cognitive process, enables individuals to selectively concentrate on relevant stimuli within complex environments while sustaining engagement over time (Posner and Rafal, 1988). During developmental years, attentional capacity serves as a cornerstone for academic success. Though often unnoticed in everyday functioning, attentional deficits become clinically significant in conditions such as Attention-Deficit/Hyperactivity Disorder (ADHD), which negatively influences learning outcomes, family relationships, and social interactions (Rodríguez et al., 2018; Adamou et al., 2013). Among the various manifestations of attention, cultivating sustained attention in children and adolescents is especially critical in the classroom, given that they spend most of their day receiving education there. The classroom thus becomes an important venue for exercising and enhancing this ability. Therefore, understanding the mechanisms and dynamics of sustained attention is particularly significant for helping doctors and cognitive rehabilitation therapists better assess, understand, and restore attention.

The Continuous Performance Test (CPT), a well-established neuropsychological paradigm requiring participants to respond rapidly to designated target stimuli while inhibiting responses to non-targets, has been extensively utilized to measure vigilance and impulsivity in sustained attention tasks (Hall et al., 2016; Rapport et al., 2000). Although CPT serves as a supplementary diagnostic tool for ADHD, learning disabilities, and depression (Voinescu et al., 2023), its clinical efficacy remains contested due to limitations in sensitivity, specificity, and ecological validity (Eom et al., 2019). These constraints have catalyzed the development of Virtual Reality-based CPT (VR-CPT), which enhances ecological relevance by replicating authentic classroom environments with multisensory distractors. VR-CPT has exhibited enhanced diagnostic precision in distinguishing ADHD from non-ADHD populations compared to traditional CPT (Rodríguez et al., 2018), and its applications have expanded to interventions for various neurodevelopmental and psychiatric disorders, including autism spectrum disorder, post-traumatic stress disorder, and schizophrenia (Cieślik et al., 2020; Park et al., 2019). Contemporary VR-CPT implementations frequently incorporate virtual classrooms enhanced with visual, auditory, or multimodal distractors to improve ecological validity (Rodríguez et al., 2018; Hong et al., 2022; Díaz-Orueta et al., 2014). However, existing research predominantly employs distractors as environmental elements rather than systematically investigating their neurocognitive effects. For instance, Hong et al. (2022) examined behavioral outcomes of distractors in ADHD populations but did not include electrophysiological analyses. Similarly, Wang et al. (2024) explored time-frequency EEG characteristics during VR-CPT tasks but overlooked nonlinear dynamics. This gap in linking complex environments to neurophysiological mechanisms is being addressed in related fields. For example, recent work by Ronca et al. (2024) successfully developed an EEG-based ‘distraction index’ to quantify drivers’ cognitive states under different road conditions, demonstrating a robust framework for assessing neurophysiological distraction mechanisms in a high-fidelity simulator.

Building on the impetus to understand distraction through neurophysiological lenses, as seen in domains like driving simulation, the present study turns to VR classrooms. Yet, within sustained attention research itself, studies have predominantly focused on behavioral outcomes (Rodríguez et al., 2018; Rostami et al., 2022). While these metrics provide valuable insights into attentional processes, there remains a critical need for more specific and precise tools to evaluate distinct aspects of attention. Electroencephalography (EEG) technology offers a non-invasive, real-time method for monitoring brain activity. Event-related potentials (ERPs), defined as voltage fluctuations time-locked to sensory, cognitive, or motor events (Naatanen, 1969), are particularly informative. P300 is a component of ERP, belonging to endogenous potentials. It is not influenced by the physical characteristics of stimuli (such as shape, size, visual, auditory), but is closely related to the mental state and attention of the subjects. This positive deflection peaking approximately 300 ms post-stimulus serves as a neural correlate of attentional resource allocation and contextual updating. Analysis of P300 characteristics (latency, amplitude) enables deeper understanding of cortical dynamics during attention tasks, thereby enhancing diagnostic and therapeutic precision. Entropy analysis of biosignals, particularly EEG, has emerged as a powerful tool for characterizing complex neurophysiological processes. As a quantitative measure of signal complexity, randomness, and system unpredictability, entropy provides unique insights into the nonlinear dynamics underlying cognitive states. Ke et al. (2014) demonstrated the superiority of sample entropy (SampEn) over traditional theta/beta power ratios in visual attention tasks, highlighting the necessity of nonlinear parameters for robust attentional assessment. Wan et al. (2021, 2023) established correlations between frontal EEG nonlinear dynamics and performance variability in sustained attention tasks, further validating entropy metrics as predictors of cognitive performance. Significant classification accuracy improvements using nonlinear feature combinations were demonstrated for identifying distinct cognitive states (Boroujeni et al., 2019). The comparative study by Cuesta–Frau et al. (2017) found that SampEn and fuzzy entropy (FuzzyEn) demonstrated superior robustness in performance comparisons among entropy metrics. Clinically, EEG entropy has been recognized as a reliable biomarker for encephalopathy diagnosis (Jacob and Nair, 2019), with empirical evidence showing its sensitivity to cognitive workload modulation (Li et al., 2016). Collectively, these advancements substantiate the methodological rationale for integrating entropy-based nonlinear analysis with ERP-P300 characterization in investigating visual distractor effects within virtual classroom environments. The proven utility of entropy metrics across diverse physiological and pathological contexts supports their application in elucidating how visual distraction modulates neural complexity during sustained attention tasks.

Extensive research has demonstrated that individuals with ADHD exhibit reduced P300 amplitudes (Marquardt et al., 2018; Hasler et al., 2016; Grane et al., 2016; Szuromi et al., 2011) and prolonged latencies compared to healthy controls (Yamamuro et al., 2016; Tsai et al., 2012). Regarding the effects of distractors on neurotypical populations, Pappalettera et al. (2024) reported that auditory distraction significantly prolongs P300 latency. Previous EEG studies on attention have predominantly focused on ADHD-neurotypical comparisons using conventional screen-based paradigms. In the context of entropy analysis, Khoshnoud et al. observed significantly decreased prefrontal entropy values in ADHD subjects (Khoshnoud et al., 2018). To date, no studies have systematically investigated distractor-induced EEG alterations through nonlinear dynamic entropy analysis. To address this research gap, a VR-CPT system implementing a Go/No-go paradigm was developed, incorporating ecologically valid visual distractors simulating classroom environments. This paradigm innovatively integrates contextual visual distraction into traditional Go/No-go tasks. EEG signals were analyzed through multidimensional approaches, including time-domain characterization (P300 peak amplitude and latency), frequency-domain analysis, and nonlinear entropy metrics (SampEn, FuzzyEn), to comprehensively evaluate the effects of visual distractors on sustained attention.

We hypothesize that visual distractors will impair behavioral performance by reducing accuracy and prolonging reaction times. At the neurophysiological level, distractors are expected to increase cognitive workload, manifesting as elevated P300 amplitudes, prolonged P300 latencies, and heightened EEG signal complexity (increased entropy values).

## 2 Methods

### 2.1 Participants

The anticipated sample size was determined based on a G*Power analysis, which we set as follows: tail = two-tailed, effect size = 0.5, *α* = 0.05, power = 0.95 (paired samples *t*-test). The suggested sample size by G*Power is 54 individuals. We recruited 66 undergraduate students (36 males and 30 females) from Tianjin Medical University as research participants, with ages ranging from 18 to 25 years old (M = 20.76, SD = 1.69). All participants completed the Adult ADHD Self-Report Scale (ASRS-v1.1) to rule out significant ADHD symptoms (scores < 14). This ensured that behavioral and EEG responses were not confounded by undiagnosed attentional deficits. Other exclusion criteria included: (a) a history of neurological diseases (including head injuries or epilepsy), mental illnesses, or drug dependence; (b) uncorrected hearing and vision impairments; (c) a history of dizziness or vertigo symptoms caused by virtual reality; (d) individuals with anxiety, depression, or learning difficulties. This study was approved by the Ethics Committee of Tianjin Medical University (approval number TMUhMEC20210201) and followed the ethical principles outlined in the 1964 Declaration of Helsinki.

### 2.2 Instruments and stimuli

In this VR-based experiment, we used an HTC VIVE VR headset to present the CPT in a virtual classroom. The computer supporting the VR system had the following specifications: NVIDIA GeForce GTX 2080Ti graphics card, i7-4790K CPU (running at 4.00 GHz), 16GB RAM, and Windows 10 Professional operating system. This configuration ensured optimal performance and compatibility for the VR-CPT test. During the EEG study, we used professional equipment including a 32-channel EEG cap (NeuroScan, Quik-Cap, United States), an EEG amplifier (NuAmps, Compumedics Neuroscan, Australia) for collecting brain electrical signals from the scalp, and conductive paste (Electro-Gel, United States) to ensure optimal conductivity between electrodes and the scalp. Additionally, we utilized two computers, one for monitoring the test and another for monitoring the EEG signal.

**Figure 1:**
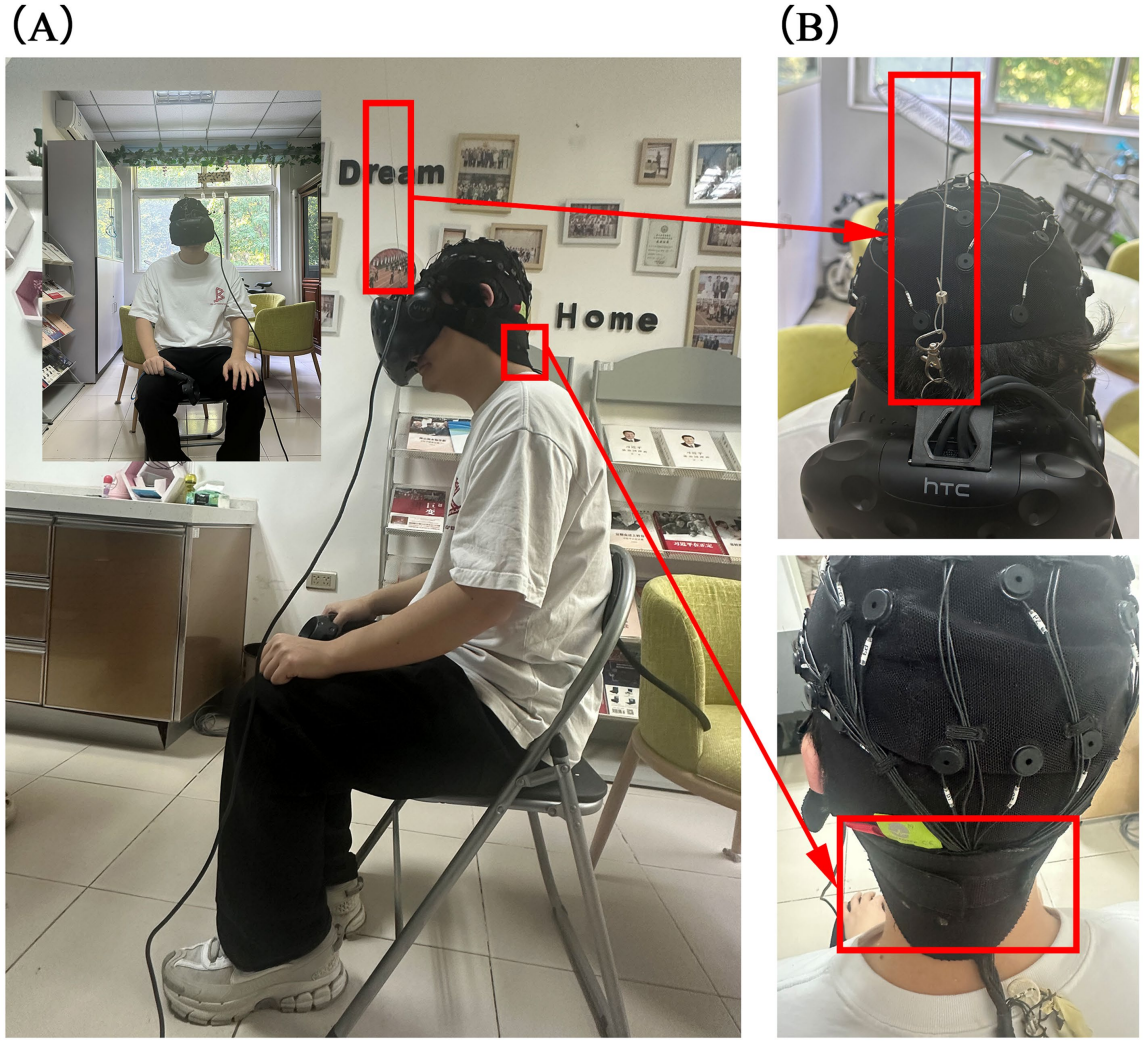
Frontal and side views of the subject and details of EEG cap placement. **(A)** The frontal and side views. **(B)** The details of EEG cap placement.

Simultaneous collection of EEG signals while performing the VR-CPT task could be affected by the weight and pressure of the headset. To address this issue, we innovatively designed a solution that suspended the weight of the VR device using spring suspension cables fixed to the ceiling of the room (as shown in Figure 1). This design significantly reduced discomfort and pressure on the brain caused by wearing the VR headset, thereby minimizing myoelectric distraction on EEG recordings.

**Figure 2:**
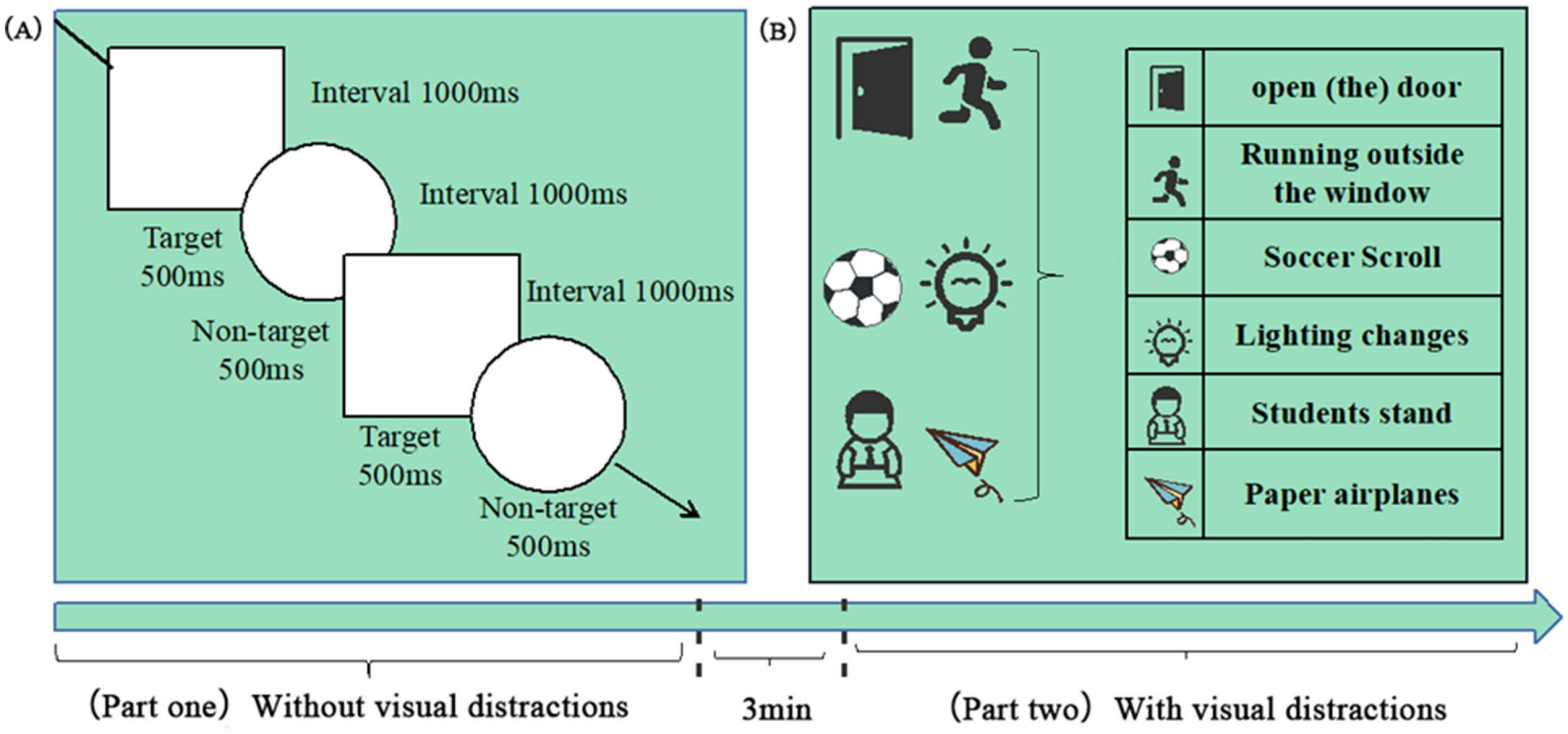
Dual-phase Go/No-go paradigm with ecological distractors. **(A)** Task structure: Participants responded to target stimuli (“□”, 100 trials) and inhibited responses to non-target stimuli (“◯”, 50 trials). **(B)** Distractor categories. Key parameters: Stimulus: 500 ms, interval: 1000 ms, and rest: 3 min between phases.

The experiment comprised two parts, which the researchers explained step-by-step. Specific instructions were as follows: participants were asked to pull the trigger of the handle when they saw a “□ “symbol on the blackboard in the virtual classroom, while ignoring the “◯” symbol. The first part had no visual distractors, whereas the second trial included visual distractors. Each trial consisted of 100 target stimuli and 50 non-target stimuli, presented randomly. Each target and non-target stimulus was displayed for 500 milliseconds, with an interstimulus interval of 1,000 milliseconds between the end of one stimulus display and the start of the next. After completing the first part, participants were given a 3-min rest before proceeding to the next trial. Figure 2 shows the experimental paradigm and the distractors used in the study.

### 2.3 VR-CPT and procedure

**Figure 3:**
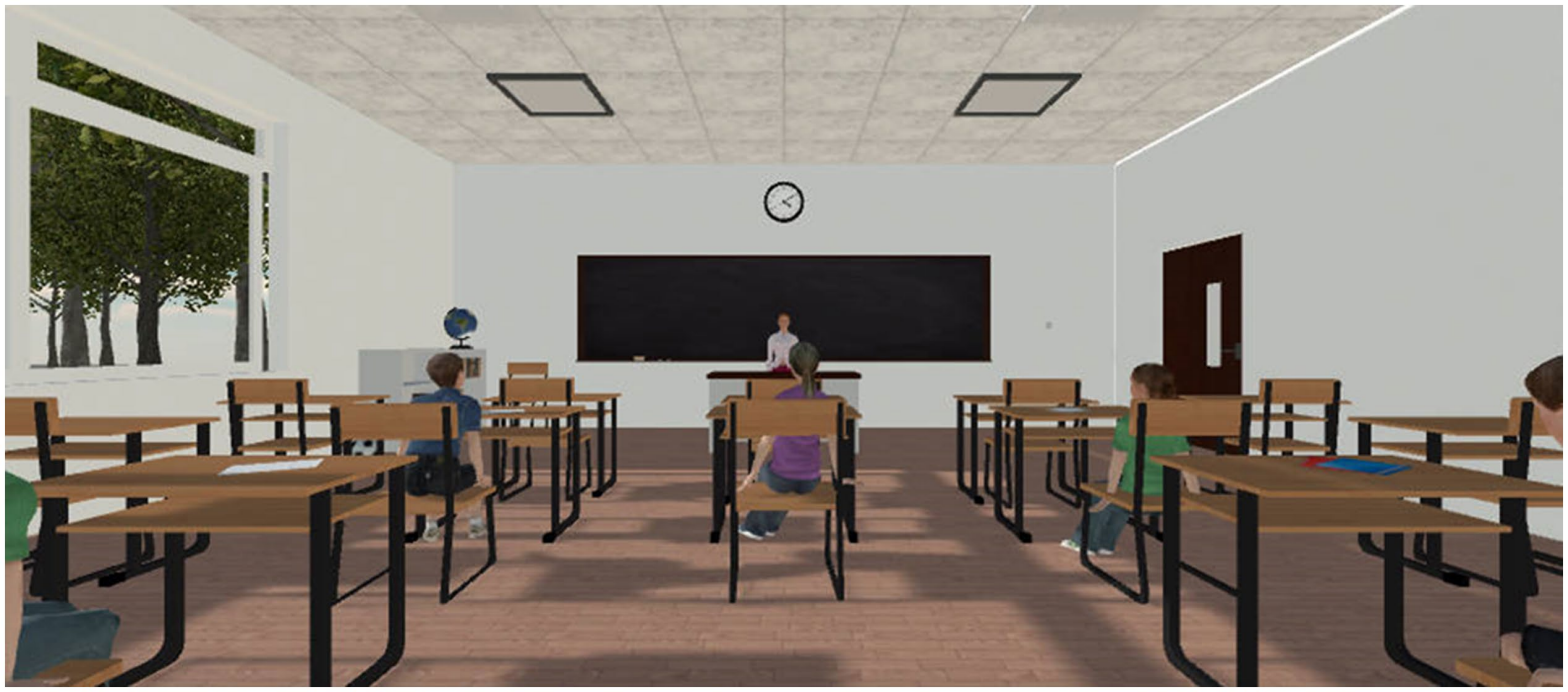
Virtual classroom VR environment.

The experimental task software was developed using Unity2021 (Unity Technologies, Inc.). The task program ran on a 17-inch monitor with a resolution of 1440 × 900 and a refresh rate of 60 Hz. Figure 3 showcases the virtual classroom scenario. The experiment included two tasks, which the researchers explained step-by-step; after completing the first task, there was a 3-min rest period before continuing to the next task.

In this experiment, participants were placed in a quiet room, seated in a comfortable chair, and instructed to concentrate and respond to visual stimuli. Prior to the experiment, each participant underwent a practice VR-CPT test. Researchers ensured participants were familiar with the VR headset and emphasized the importance of reporting any discomfort promptly. Researchers assisted participants in adjusting the VR headset to ensure comfort and optimal image focus. During the practice phase, participants became familiar with the VR environment and practiced using the controller. After the practice session, the formal experiment began. Participants had the right to withdraw at any time during the experiment.

### 2.4 Electrophysiological recording and preprocessing

**Figure 4:**
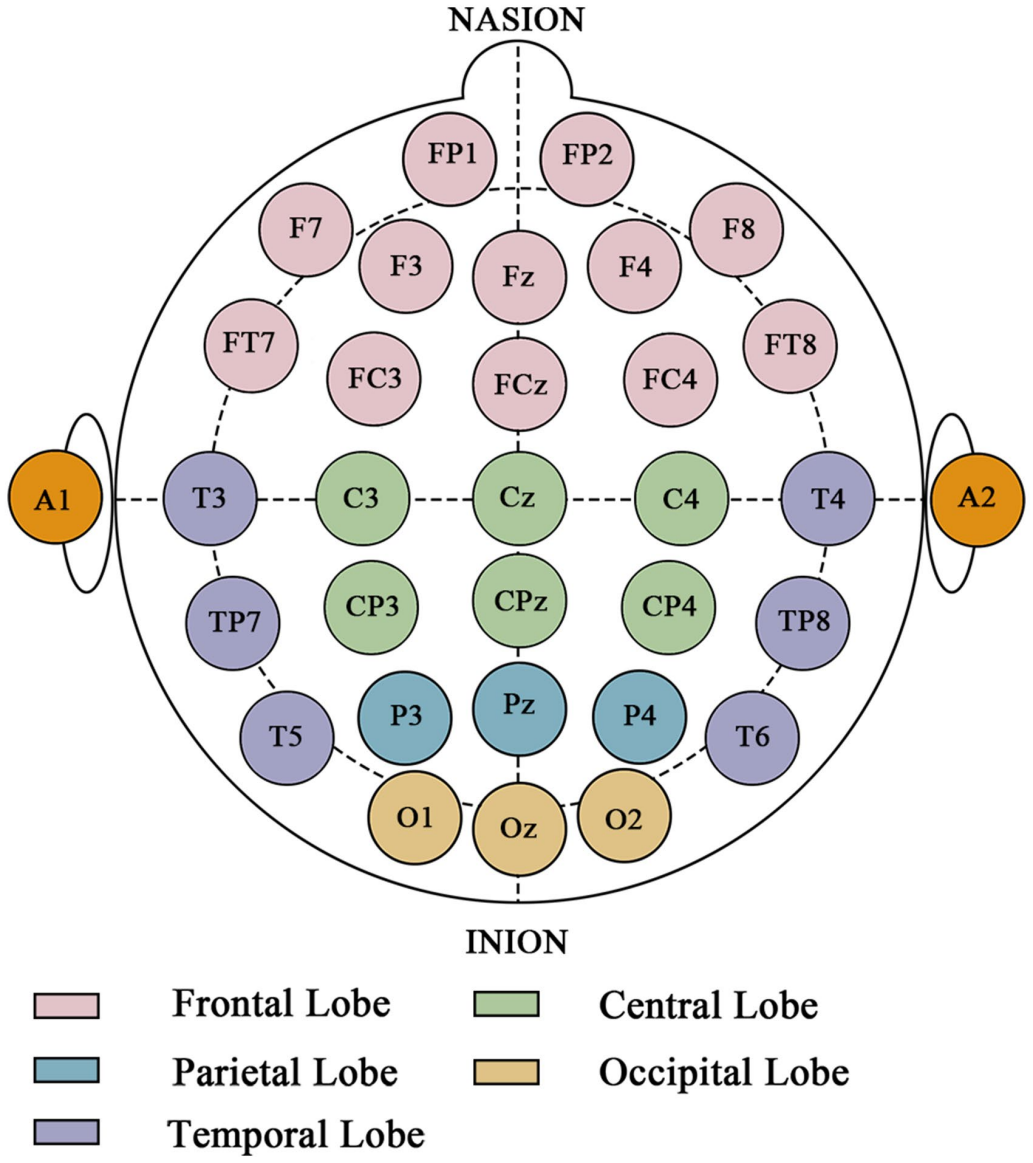
Electrode montage and cortical parcellation under extended international 10–20 system.

For this study, the EEG cap electrodes were arranged according to the extended international 10–20 system, comprising 32 electrodes. The inclusion of additional electrodes (e.g., FT7/FT8, TP7/TP8) beyond the minimal 10–20 setup allows for finer topographic analysis of neural activity. Figure 4 shows the distribution of the EEG electrodes. These included 32 fixed wired electrodes (FP1, FP2, F7, F3, Fz, F4, F8, FT7, FC3, FCz, FC4, FT8, T3, C3, Cz, C4, T4, TP7, CP3, CPz, CP4, TP8, T5, P3, P4, Pz, T6, A1, A2, O1, O2 and Oz). The left mastoid electrode serves as the reference electrode and the Fpz electrode serves as the ground electrode. Conductive gel was used as the medium between the electrodes and the scalp, and all of the electrodes were sintered Ag/AgCl electrodes. During data acquisition, the electrode impedance was kept below 5 kΩ at a sampling rate of 1,000 Hz. The brain was divided into five regions, as shown in Figure 4. The frontal lobe includes the Fp1, Fp2, F7, F3, Fz, F4, F8, FT7, FC3, FCz, FC4 and FT8 electrodes. The central lobe includes the C3, Cz, C4, CP3, CPz and CP4 electrodes. The parietal lobe includes the P3, Pz and P4 electrodes. The occipital lobe includes the O1, Oz and O2 electrodes. The temporal lobe includes the T3, TP7, T5, T4, TP8 and T6 electrodes.

**Figure 5:**
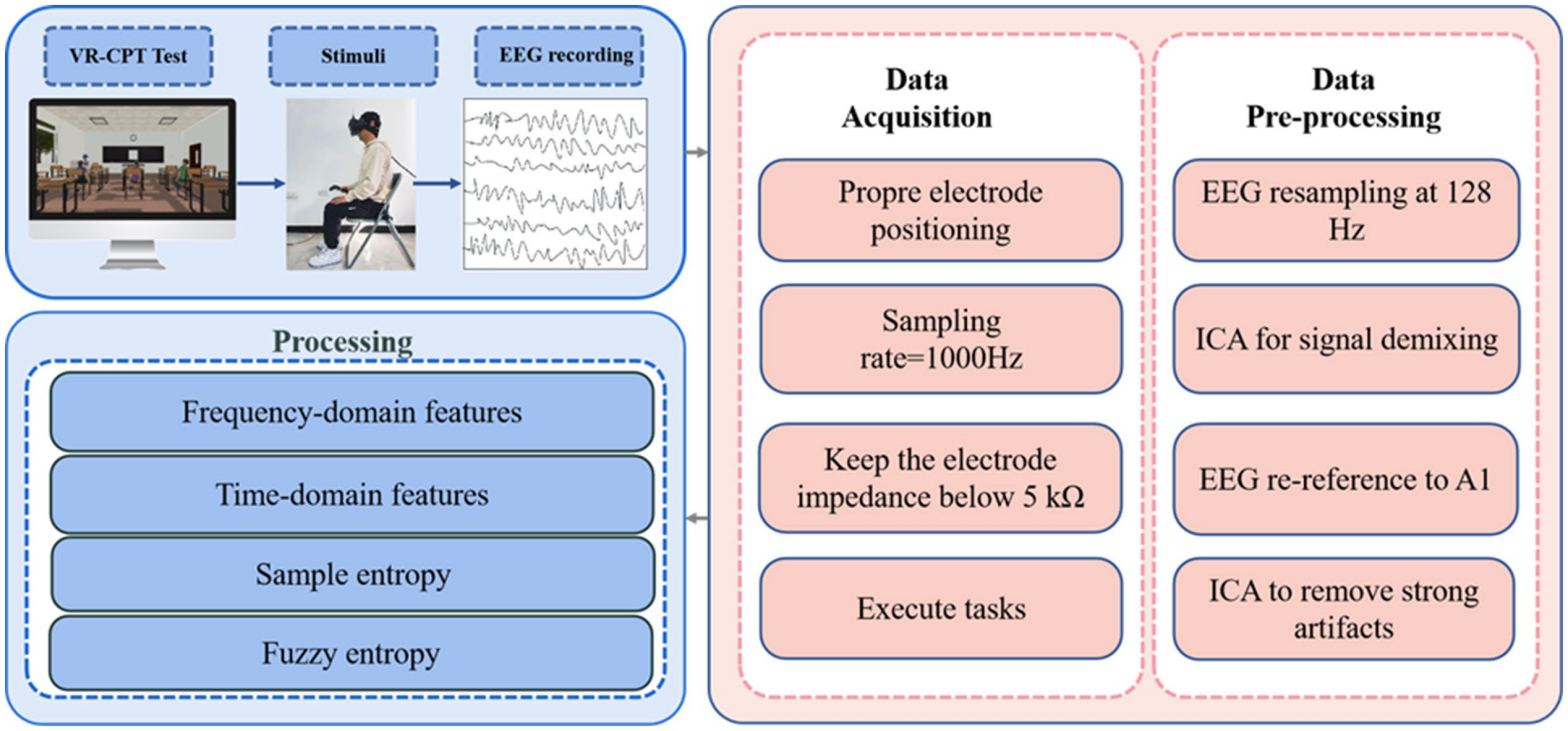
Virtual reality continuous performance test EEG data processing workflow diagram.

Due to the susceptibility of EEG data to noise and artifacts, preprocessing is crucial before analysis. Using the EEGLAB toolbox (version 13.6.5b) on the MATLAB platform, the EEG data were preprocessed, including applying a bandpass filter from 0.5 Hz to 30 Hz (Delta waves: 0.5–4 Hz, Theta waves: 4–8 Hz, Alpha waves: 8–12 Hz, Beta waves: 12–30 Hz). Following band-pass filtering, artifact correction was performed using a two-stage process. First, Independent Component Analysis (ICA) was applied to the continuous data to separate source components. Second, the resulting ICA components were independently reviewed and classified by two experienced EEG analysts who were blinded to the experimental conditions. The classification was based on the components’ characteristic features in the time domain, frequency domain, and scalp topography (Figure 5). Components identified as artifacts by both analysts were removed. In cases of disagreement, a consensus was reached through discussion. The remaining components, judged to be of cerebral origin, were back-projected to reconstruct the clean, continuous EEG signal for all subsequent analyses. To assess consistency, inter-rater reliability was calculated on a randomly selected 20% subset of the data using Cohen’s kappa, yielding a substantial agreement (*κ* = 0.78). Subsequently, the EEG data were segmented into 1,000 millisecond epochs (200 milliseconds before each stimulus target and 800 milliseconds after each stimulus target). These epochs were baseline-corrected using the pre-stimulus interval (−200 to 0 ms). For the entropy analysis, SampEn and FuzzyEn were calculated for the post-stimulus interval (0–800 ms) of each epoch and then averaged for each participant and condition. The entropy metrics were computed for each electrode separately, without global normalization across channels, to preserve the local complexity information at each scalp site. In MATLAB, the “spectrogram” function based on short-time Fourier transform (STFT) with a Hanning window (window size: 200 milliseconds) was mainly used to calculate the power spectral density of the EEG signal.

SampEn is a method for measuring the complexity of time series proposed by Richman (Richman and Moorman, 2000). It is generally denoted as SampEn (*m, r, N*), where *r* is the similarity tolerance, *N* is the length, and *m* is the dimension. The physical meaning of SampEn is to represent the rate at which a nonlinear dynamical system generates new information; the larger the SampEn value, the more complex the sequence.

Generally, for a time series composed of *N* data points (Equation 1):


(1) {x(n)}=x(1),x(2),x(3)⋯x(N)

The specific algorithm for SampEn is as follows:

(1) Form a group of m-dimensional vectors in sequential order by index (Equation 2):

(2) X(i)=[x(i),x(i+1),⋯,x(i+m−1)],i=1,2,3⋯,N−m+1

(2) Define the distance between X(i) and X(j) as the maximum difference between corresponding elements (Equation 3).

(3) $$d[X(i),X(j)]=\overset{m-1}{\max_{k=0}}|x(i+k)-x(j+k)|;1\leq j\leq N-m+1,j\neq i$$

(3) Set a similarity tolerance threshold *r* (*r* > 0). For each value of i≤N−m , count the number of d[X(i),X(j)] value less than *r*, and calculate the ratio of this number to the total number of distances *N − m + 1*, denoted as $B_{i}^{m}(r)$ (Equation 4).

(4) $$\begin{array}{l} B_{i}^{m}(r)=\frac{1}{N-m-1}\\ \left\{\text{Number of pairs}\;(j)\;\text{satisfying}\;d[X(i),X(j)]<r\right\} \end{array}$$

(4) Calculate the average value $\;B_{i}^{m}(r)$ for al li (Equation 5):

(5) $$B^{m}(r)=\frac{1}{N-m}\sum_{i=1}^{N-m}B_{i}^{m}(r)$$

(5) Increase the dimension to *m + 1* and repeat steps (1) to (4) to obtain $B^{m+1}(r)$ (Equation 6).

(6) $$B^{m+1}(r)=\frac{1}{N-m}\sum_{i=1}^{N-m}B_{i}^{m+1}(r)$$

Theoretically, the SampEn of this sequence is (Equation 7):


(7) $$\text{SampEn}(m,r,N)=\lim_{N\to \infty }[-\ln\frac{B^{m+1}(r)}{B^{m}(r)}]$$

When *N* is a finite value, the SampEn of a finite-length sequence is (Equation 8):


(8) $$\text{SampEn}(m,r,N)=-\ln[\frac{B^{m+1}(r)}{B^{m}(r)}]$$

The value of SampEn is closely related to the dimension *m* of the data segment and the similarity tolerance *r*. Different values of m and r correspond to different values of SampEn, so the selection of SampEn parameters is particularly important. Currently, the values for the dimension of the data segment and the similarity tolerance are generally chosen based on empirical values. In the calculation of SampEn, the dimension of the data segment m is typically set to 2–10, with the actual dimension determined by the specific circumstances. The similarity tolerance r is generally set to 0.1–0.5 times the standard deviation of the data segment (Chen et al., 2018). In this study, the data segment dimension *m = 2* and similarity tolerance *r = 0.25* standard deviation were selected for SampEn feature extraction.

FuzzyEn is an improvement on the SampEn algorithm proposed by Chen et al., which introduces fuzzy membership functions as a measure of time series complexity (Chen et al., 2007). Its physical significance represents the rate at which a nonlinear dynamic system generates new information; the smaller the FuzzyEn value, the higher the self-similarity of the sequence; the larger the FuzzyEn value, the more complex the sequence. Unlike SampEn, FuzzyEn does not use bimodal classification when measuring shape similarity but instead uses fuzzy functions to reflect sample similarity, which is more advantageous when dealing with nonlinear and non-smooth signals.

The definition algorithm for FuzzyEn is as follows:

(1) N-point sampled sequence (Equation 9):

(9) {x(n)}=x(1),x(2),x(3)⋯x(N)

(2) Define $U_{m}^{i}$ represents m consecutive x values as follows (Equation 10):

(10) $$\begin{array}{l} U_{m}^{i}=[x(i),x(i+1),\cdots ,x(i+m-1)]-\bar{x(i)},\\ i=1,2,\cdots ,N-m+1\;\; \end{array}$$

where $\bar{\;x(i)}\;$ represents the local mean over the embedding window (Equation 11):


(11) $$\bar{x(i)}=\frac{1}{m}\sum_{j=0}^{m-1}x(i+j)$$

(3) Define the distance $d_{\mathrm{ij}}^{m}$ between the vectors $X_{i}^{m}$ and $X_{j}^{m}$ as the maximum absolute difference of the corresponding scalar components (Equation 12):

(12) $$d_{\mathrm{ij}}^{m}=d[X_{i}^{m},X_{j}^{m}]=\max_{k\in (0,m-1)}\{|x(i+k)-\bar{x(i)}-(u(j+k)-\bar{x(j)})|\}$$

(4) Calculate the similarity degree $D_{\mathrm{ij}}^{m}$ between the vectors $X_{i}^{m}$ and $X_{j}^{m}$ through a fuzzy membership function $\mu (d_{\mathrm{ij}}^{m},n,r)$ (Equation 13):

(13) $$D_{\mathrm{ij}}^{m}(n,r)=\mu (d_{\mathrm{ij}}^{m},n,r)$$

(5) where the fuzzy membership function $\mu (d_{\mathrm{ij}}^{m},n,r)$ is an exponential function, r is the similarity tolerance, and n is the fuzzy index, respectively (Equation 14):

(14) $$\mu (d_{\mathrm{ij}}^{m},n,r)=\exp(-\frac{{\left(d_{\mathrm{ij}}^{m}\right)}^{n}}{r})$$

(6) Define the function $\Phi^{m}(n,r)$ (Equation 15)

(15) $$\Phi^{m}(n,r)=\frac{1}{N-m}\sum_{i=1}^{N-m}(\frac{1}{N-m-1}\sum_{j=1,j\neq i}^{N-m}D_{\mathrm{ij}}^{m})$$

(7) Repeat (1)-(6) to construct the function $\Phi^{m+1}(n,r)$ (Equation 16)

(16) $$\Phi^{m+1}(n,r)=\frac{1}{N-m}\sum_{i=1}^{N-m}(\frac{1}{N-m-1}\sum_{j=1,j\neq i}^{N-m}D_{\mathrm{ij}}^{m+1})$$

(8) Define the FuzzyEn of the sequence as the negative natural logarithm of the deviation of $\Phi^{m}(n,r)$ from $\Phi^{m+1}(n,r)$ (Equation 17)

(17) $$\text{FuzzyEn}(m,n,r)=\lim_{N\to \infty }[\ln\Phi^{m}(n,r)-\ln\Phi^{m+1}(n,r)]$$

A finite data set can be approximated using (Equation 18)


(18) $$\text{FuzzyEn}(m,n,r,N)=\ln\Phi^{m}(n,r)-\ln\Phi^{m+1}(n,r)$$

where *m* and *r* are the embedding dimensions of the phase space and similarity tolerance, respectively. The parameter m determines whether the dynamic evolution of the system is reconstructed with finer granularity, while the similarity tolerance *r* governs sensitivity to subtle variations. A higher similarity tolerance may result in information loss, whereas underestimating *r* significantly increases sensitivity to noise (Lin et al., 2017). In this study, we selected *m* = 2, *r* = 0.2 × *SD*, and *n* = 2.

### 2.5 Statistical analyses

This study examined the effects of visual distraction on both behavioral and physiological signals during VR-CPT tasks, which were divided into two groups according to the nature of the trial: a visual trial without visual distraction (Group N-D) and a visual trial with visual distraction (Group Y-D). Regression analysis was conducted using the Bootstrap method, employing 95% bias-corrected Bootstrap confidence intervals (CIs) with 10,000 resampling iterations. This approach provides more robust CIs and probability values for regression model estimates, addressing issues associated with non-normal distributions (Fox, 2015). Demographic data and behavioral data, along with peak amplitudes and latencies of ERP-P300 extracted from EEG, were processed using SPSS 21.0 (IBM Corp., Armonk, NY, United States). All data analyzed with SPSS 21.0 were visually inspected through Q-Q plots and histograms. In some instances, the data exhibited non-normal distributions, yet no severe violations were observed. For behavioral data and the peak amplitudes and latencies of ERP-P300 extracted from EEG, paired *t*-tests were conducted. Given the robustness of the paired sample *t*-test to non-normality, Mann–Whitney U tests were used for non-normally distributed data.

## 3 Result

### 3.1 Behavioral results

**Figure 6:**
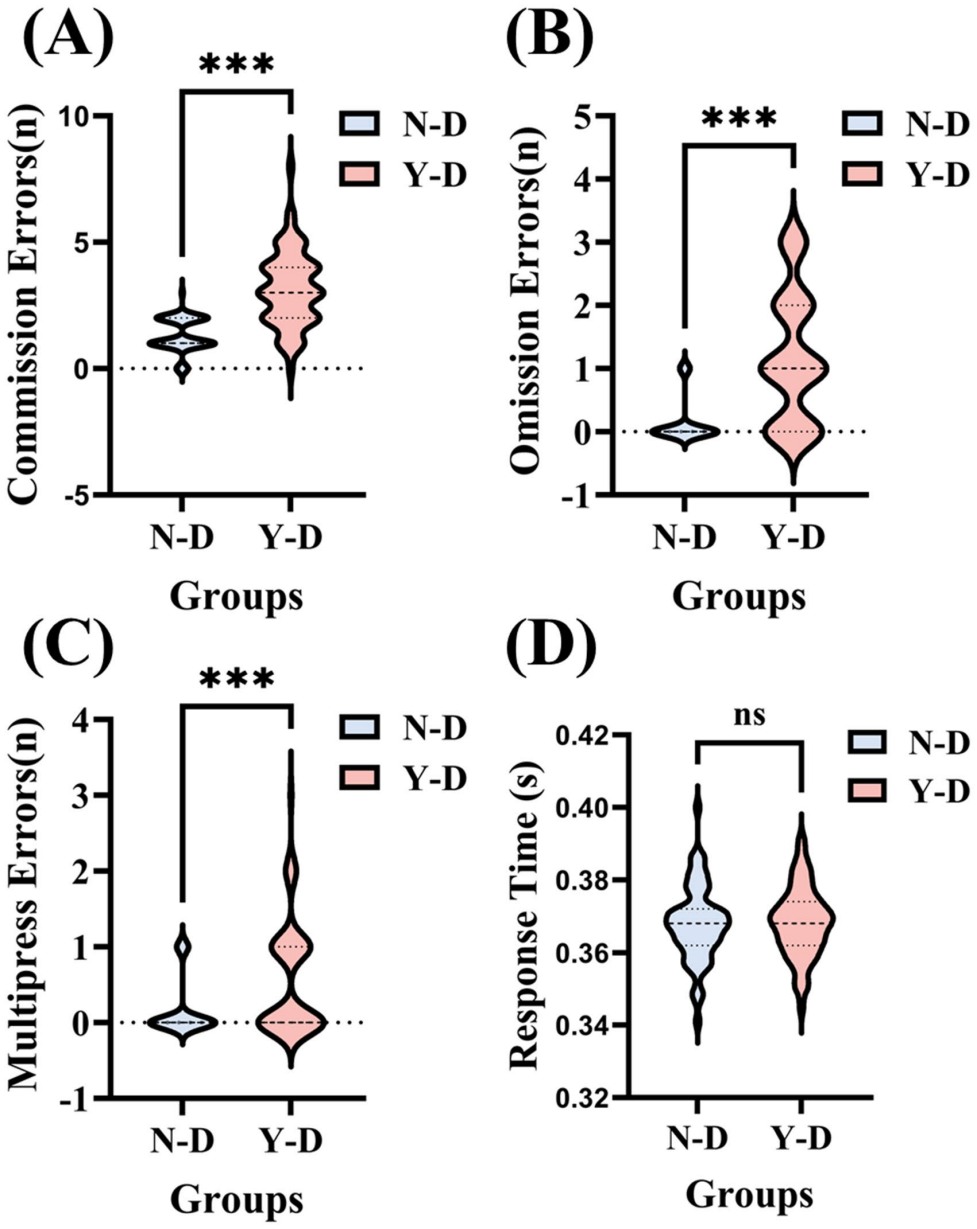
Systematic effects of visual distractors on sustained attentional performance across four behavioral dimensions. **(A)** Commission errors: Incorrect responses to non-targets. **(B)** Omission errors: missed responses to targets. **(C)** Multipress errors: double responses to targets. **(D)** Response time: target response latency. Significance levels are denoted as ***p < 0.001, **p < 0.01, *p < 0.05. Condition coding: N-D: no distractor condition and Y-D: visual distractor condition.

The results of the virtual classroom task served as a measure of sustained attention, focusing on the number of commission errors (incorrect responses to non-target stimuli), omissions (failure to respond to target stimuli), multipresses (pressing twice for a target stimulus), and response times. Paired-sample *t*-tests were conducted on the behavioral results of 66 participants. As shown in Figure 6, for the number of commission errors, the average number of errors under no visual distraction (N-D) was 1.33, while under visual distraction (Y-D), the average number of errors significantly increased to 3.15. The standard deviation indicates considerable individual differences within both groups, with more variability observed under Y-D conditions.

A paired-sample *t*-test revealed a significant difference in the number of errors between N-D and Y-D (*t* = −8.736, df = 65, *p* < 0.001), indicating that distractors significantly interfered with the participants’ attention. In terms of omissions, the average number of omissions under N-D was 0.14, which increased to 1.18 under Y-D. Standard deviations showed considerable individual differences within both groups. A paired-sample *t*-test indicated a significant difference in the number of omissions between N-D and Y-D (*t* = −8.134, df = 65, *p* < 0.001), suggesting that distractors also significantly increased the participants’ omissions. This could be because their attention was drawn to the distractors, preventing them from fully attending to or remembering all necessary information. For multipress, the average number of double-presses under N-D was 0.15, compared to 0.56 under Y-D. Despite large standard deviations within both groups, a paired-sample *t*-test showed a significant difference in the number of double-presses between N-D and Y-D (*t* = −4.598, df = 65, *p* < 0.001), indicating that distractors may have caused participants to engage in unnecessary distractions or impulsive behaviors during the task. These extraneous actions could be due to lack of concentration or cognitive disarray, further demonstrating the distraction of distractors with participant attention. Regarding response times, the average response time under N-D was 0.367 s, while under Y-D, it was 0.368 s. The standard deviations were small in both groups, indicating minimal individual differences in response times. A paired-sample *t*-test showed no significant difference in response times between N-D and Y-D (*t* = −0.200, df = 65, *p* = 0.842), indicating that the presence of distractors did not significantly affect participant response times. Although no significant difference in reaction times was observed between the distraction and non-distraction conditions, this may be attributable to the task design. Specifically, the fixed interstimulus interval of 1,000 milliseconds may have allowed sufficient processing time between stimuli, thereby reducing temporal variability and dampening potential effects of distraction on response latency.

### 3.2 EEG result analysis

**Figure 7:**
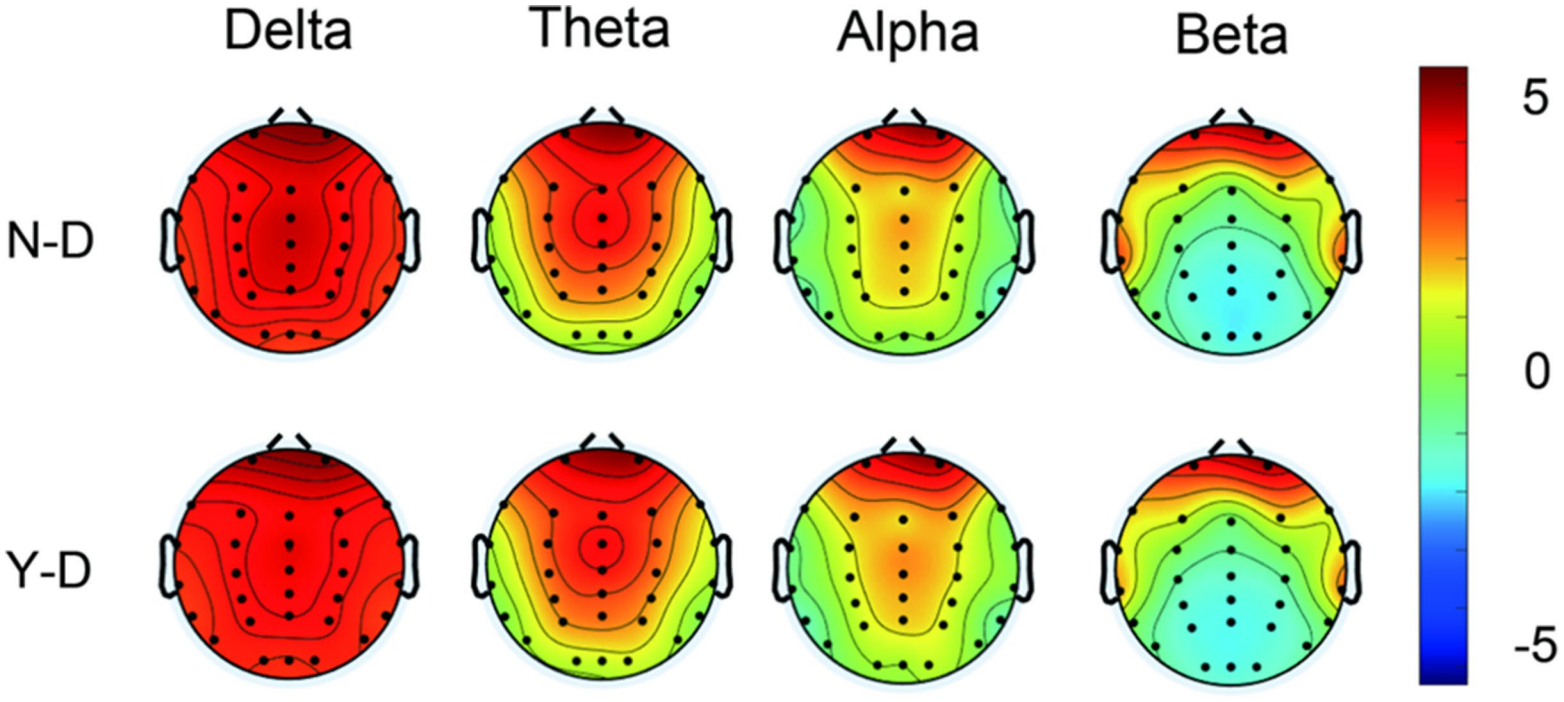
Topographical mapping of spectral power in delta/theta/alpha/beta frequency bands contrasting N-D and Y-D groups.

We utilized the short-time Fourier transform algorithm for power spectral analysis. Figure 7 displays the brain maps of the two groups in the Delta, Theta, Alpha, and Beta frequency bands. We observed that the Delta band was widely distributed across all regions in both groups. It is noteworthy that the overall spectral power was dominated by Delta band activity in both groups, with pronounced amplitudes over the frontal regions. While elevated Delta power is often associated with drowsiness or sleep, a growing body of research suggests that in the context of demanding cognitive tasks requiring sustained attention and continuous inhibitory control, increased frontal Delta oscillations may reflect an active neural mechanism for top-down inhibition and cognitive effort (Harmony, 2013). Therefore, we interpret this pattern not as a state of reduced consciousness, but rather as a marker of engaged neural processing specific to the high cognitive load of our task. The Theta band activity was primarily located in the frontal, central, and parietal regions in both groups. Alpha band activity was mainly concentrated in the frontal region, with some distribution in the central and parietal areas. Beta band activity was predominantly found in the frontal region. Upon examining the brain maps, we noted similar patterns of activity distribution in the frontal regions across groups, however, differences were observed in the central and parietal regions. Substantial literature has established that the P300 component, particularly the P3b subcomponent associated with context updating and attentional allocation, exhibits a characteristic scalp distribution that is maximal along the midline, typically peaking at centro-parietal sites (Polich, 2007). Consequently, we selected electrodes Fz, FCz, Cz, CPz, Pz, and Oz for focused in-depth analysis.

### 3.3 ERP-P300 components analysis

**Figure 8:**
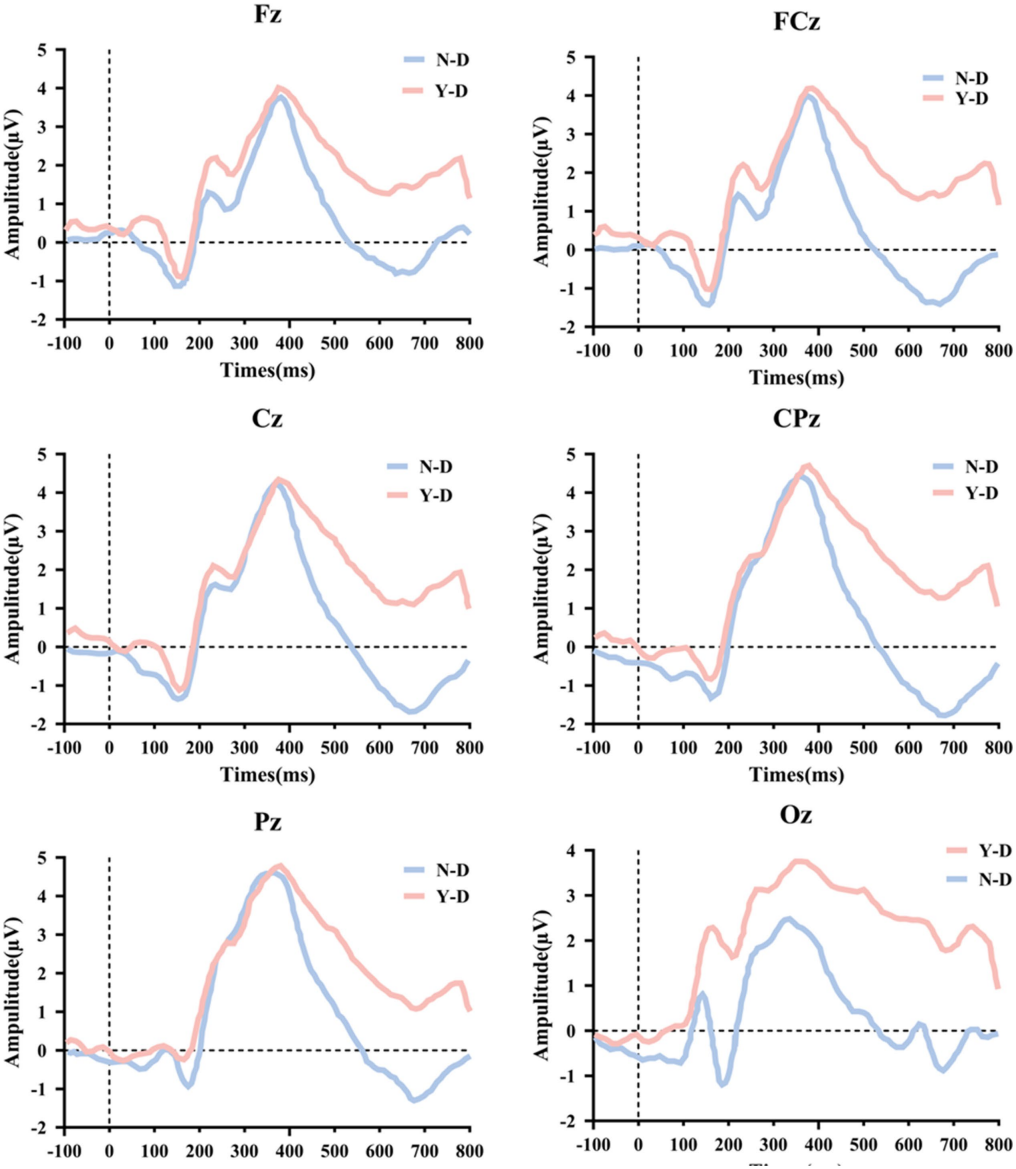
Topographic alterations of P300 peak and latency under visual distraction across parieto-occipital electrodes.

**Figure 9:**
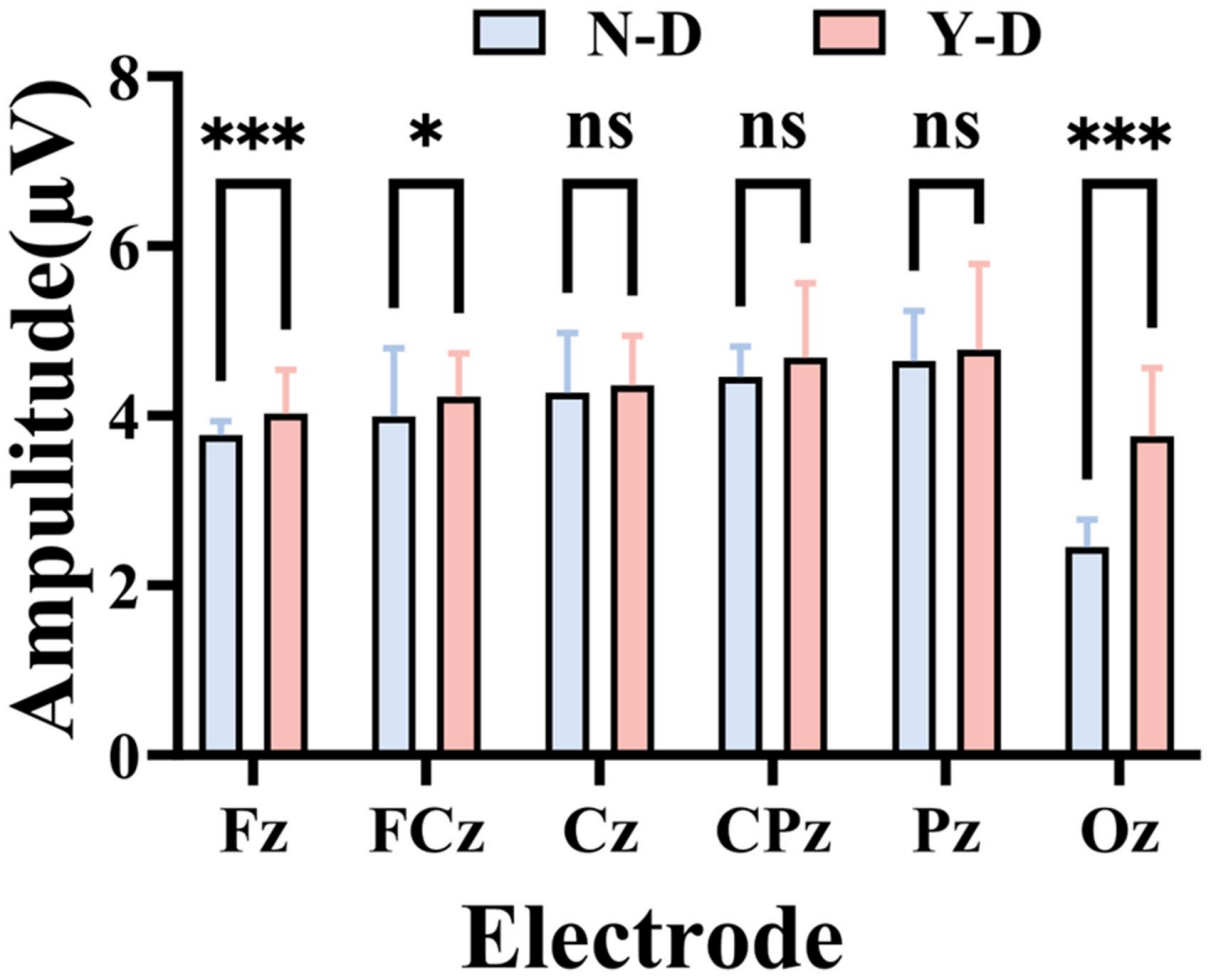
Statistically validated P300 latency prolongation under visual distraction at six critical electrode sites. Significance levels are denoted as ***p < 0.001, **p < 0.01, and *p < 0.05.

Time domain analysis was deployed to investigate the peak and latency of the P300 in ERP. Firstly, Figure 8 illustrates the ERP waveforms for six electrodes: CPz, Pz, Cz, FCz, Fz, and Oz. From these images, it is evident that the addition of visual distractors significantly increased the cognitive load on the brain. Subsequently, a paired-sample *t*-test was used to compare the latency of the P300 component between the N-D and Y-D conditions at these six electrode locations (Figure 9). The results indicate that the presence of distractors significantly increased the latency of the P300 across multiple electrode sites. Specifically, at the CPz electrode site, the average latency increased from 361.69 ms under N-D to 377.10 ms under Y-D, showing a significant delay [t(65) = −5.420, *p* < 0.001]. Similarly, at the PZ electrode site, the average latency rose from 356.09 ms to 376.06 ms [t(65) = −5.314, *p* < 0.001]. At the Cz electrode site, the latency increased from 367.74 ms to 380.38 ms [t(65) = −2.646, *p* < 0.05]. At the FCz electrode site, the latency went from 375.82 ms to 381.99 ms [t(65) = −2.100, *p* < 0.05], while at the Fz electrode site, the latency changed from 377.73 ms to 380.17 ms, with a difference approaching significance [t(65) = −1.777, *p* = 0.080]. The most pronounced effect was observed at the Oz electrode site, where the average latency increased from 327.94 ms to 351.42 ms [t(65) = −5.720, *p* < 0.001]. These findings suggest that the presence of distractors delayed cognitive processing as indicated by the P300 latency, with the most significant effects observed at the CPz, Pz, and Oz electrode sites.

**Figure 10:**
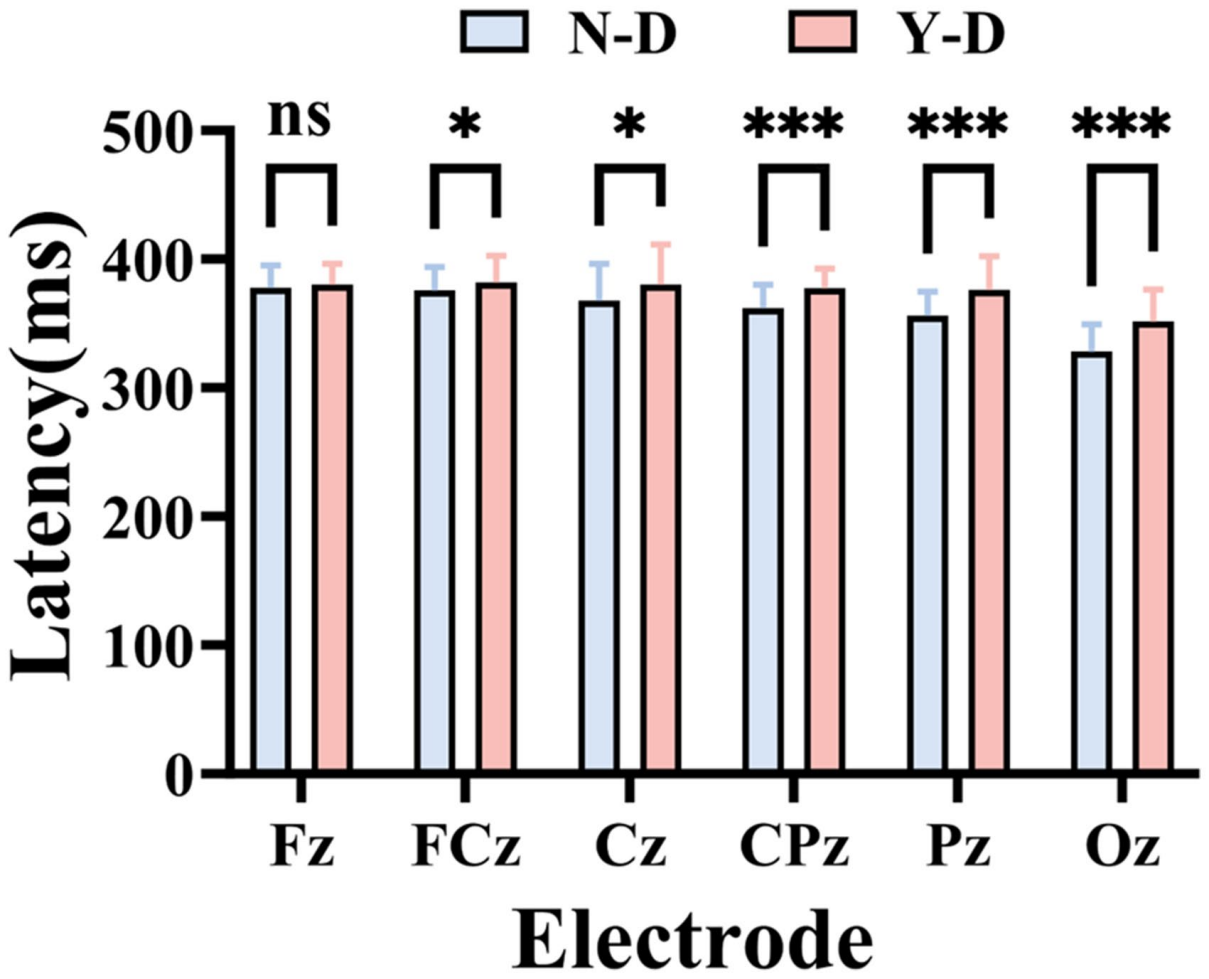
Statistically validated P300 amplitude peak under visual distraction at six critical electrode sites. Significance levels are denoted as ***p < 0.001, **p < 0.01, and *p < 0.05.

Finally, the EEG technique was used to measure the peak amplitude of the ERP-P300 component at six different electrode locations (Figure 10), and the data under the N-D and Y-D conditions were compared. At the Fz electrode location, the average P300 peak amplitude under N-D was 3.772 μV (SD = 0.161 μV), while under Y-D it was 4.024 μV (SD = 0.518 μV). A paired-sample *t*-test showed a significant difference between the two conditions (*p* < 0.001). At the FCz electrode location, the average P300 peak amplitude under N-D was 3.993 μV (SD = 0.805 μV), and under Y-D it was 4.221 μV (SD = 0.518 μV). A paired-sample *t*-test indicated a significant difference between the two conditions (*p* = 0.031). At the Cz electrode location, the average P300 peak amplitude under N-D was 4.268 μV (SD = 0.710 μV), and under Y-D it was 4.354 μV (SD = 0.586 μV). A paired-sample *t*-test showed that the difference between the two conditions was not significant (*p* = 0.381). At the CPz electrode location, the average P300 peak amplitude under N-D was 4.468 μV (SD = 0.345 μV), and under Y-D it was 4.573 μV (SD = 0.775 μV). A paired-sample *t*-test revealed that the difference between the two conditions was not significant [t(65) = −0.891, *p* = 0.377]. At the Pz electrode location, the average P300 peak amplitude under N-D was 4.640 μV (SD = 0.597 μV), and under Y-D it was 4.778 μV (SD = 1.012 μV). A paired-sample *t*-test indicated that the difference between the two conditions was not significant [t(65) = −0.983, *p* = 0.329]. At the Oz electrode location, the average P300 peak amplitude in the absence of distractors (N-D) was 2.453 μV (standard deviation = 0.326 μV), and in the presence of distractors (Y-D) it was 3.758 μV (SD = 0.805 μV). A paired-sample *t*-test showed a significant difference between the two conditions (*p* < 0.001). Figure 10 presents the statistical results of the peak values for the ERP-P300 component.

This finding suggests that when designing attention testing systems, considering the differential effects of distractors on various brain regions can help optimize the system’s detection and response to divided attention states. By accurately identifying and analyzing changes in P300 peak amplitude, the system can better manage attentional resources, improving cognitive performance and user experience in complex environments.

### 3.4 Analysis of entropy in EEG

This study analyzed EEG signals based on SampEn and FuzzyEn. Through significance testing of 30 regional electrodes, significant spatial heterogeneity was observed in the responses of different brain regions to distracting stimuli, providing data-driven insights into the neural mechanisms of attentional regulation.

**Figure 11:**
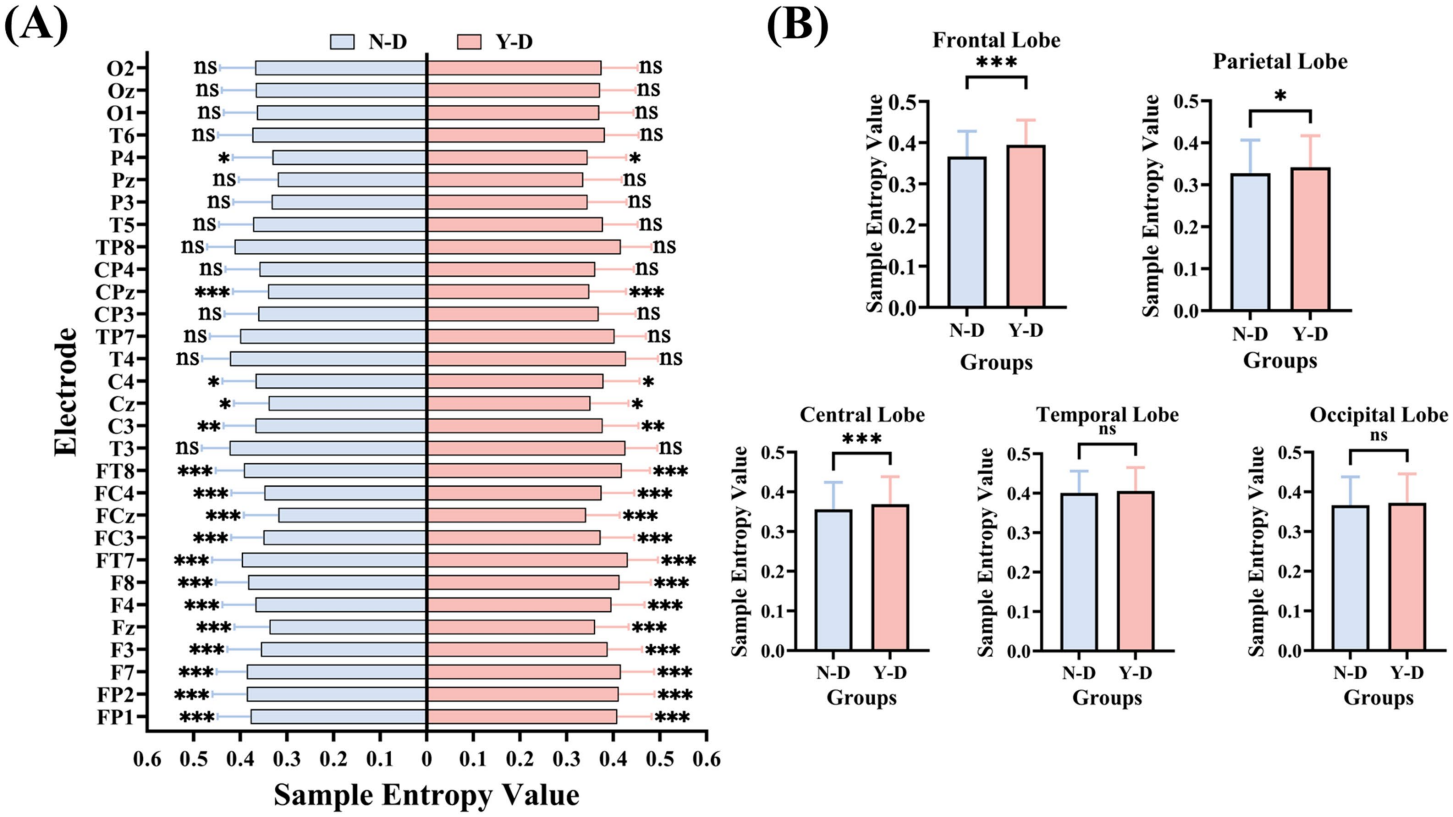
Brain-region specific modulation of neural complexity by visual distraction measured by sample entropy. **(A)** Electrode-wise comparison. **(B)** Lobar-level integration. Significance levels are denoted as ***p < 0.001, **p < 0.01, *p < 0.05.

The results of SampEn (as shown in Figure 11) revealed the following: Frontal electrodes (FP1, FP2, F7, F3, Fz, F4, F8, FT7, FC3, FCz, FC4, and FT8) exhibited significantly higher SampEn values under distraction conditions compared to non-distraction conditions. Central electrodes (C3, Cz, C4, CPz) showed significant increases in SampEn during distraction. Parietal electrode P4 demonstrated significantly elevated SampEn under distraction. No significant differences were observed in other electrodes between distraction and non-distraction conditions.

**Figure 12:**
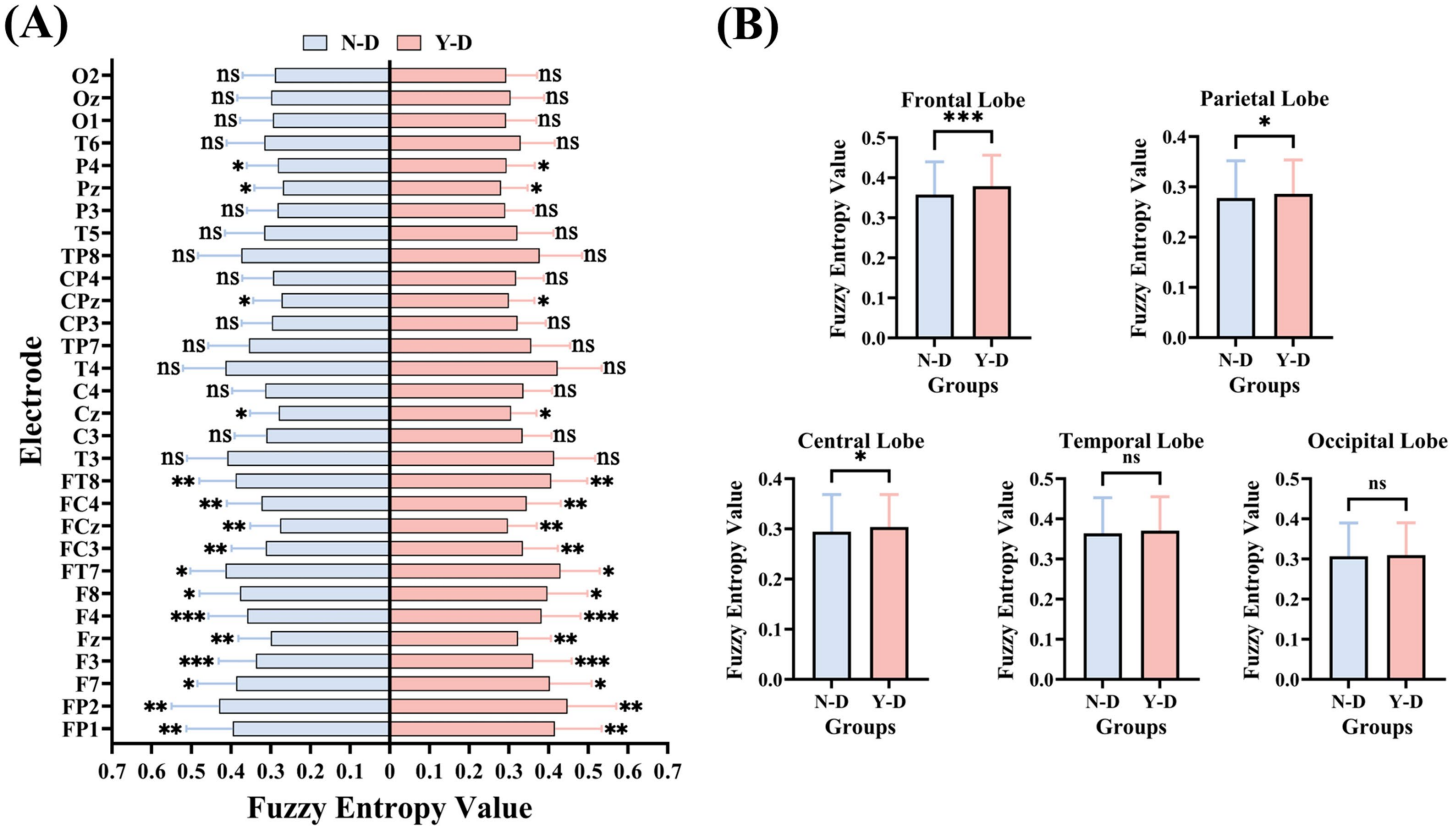
Brain-region specific modulation of neural complexity by visual distraction measured by fuzzy entropy. **(A)** Electrode-wise comparison. **(B)** Lobar-Level Integration. Significance levels are denoted as ***p < 0.001, **p < 0.01, *p < 0.05.

The results of FuzzyEn (as shown in Figure 12) indicate that the frontal electrodes FP1, FP2, F7, F3, Fz, F4, F8, FT7, FC3, FCz, FC4, and FT8 exhibited significant differences in FuzzyEn values between distraction and non-distraction conditions, with higher values under distraction. Central electrodes Cz and CPz showed significant differences in FuzzyEn between distraction and non-distraction conditions, with higher values during Y-D. Parietal electrodes P4 and Pz demonstrated significant differences in FuzzyEn between conditions, with higher values under Y-D. No significant differences in FuzzyEn were observed for other electrodes.

**Figure 13:**
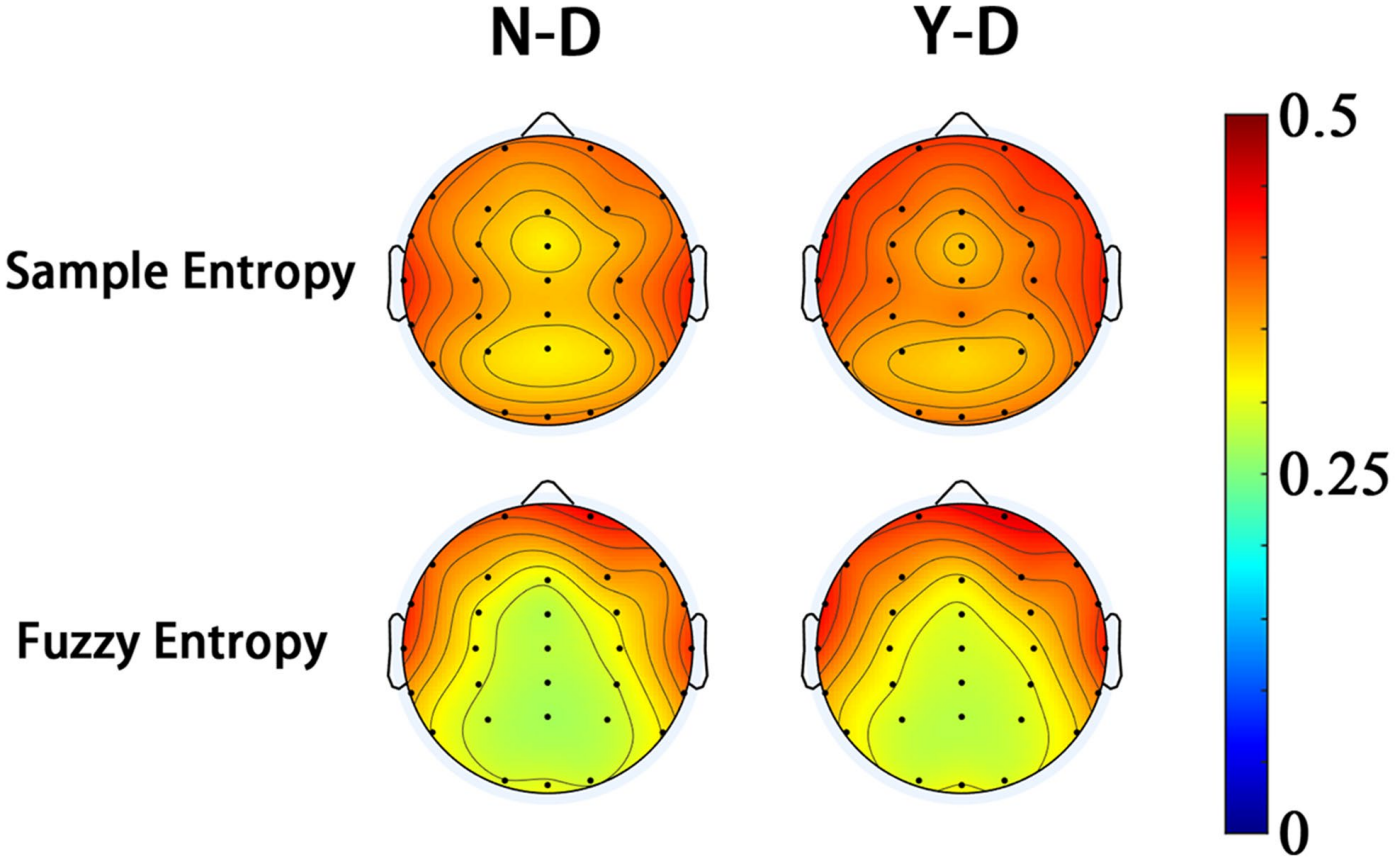
Topographic mapping of entropy metrics reveals fronto-central-parietal enhancement under visual distraction.

Analysis of SampEn and FuzzyEn across brain regions (as shown in Figure 13) revealed that both metrics exhibited higher values in the frontal, central, and parietal regions under distraction conditions in the Y-D and N-D groups, as visualized in their respective topographic maps.

The SampEn results (as shown in Figure 11) demonstrated significant differences between Y-D and N-D conditions in the frontal, central, and parietal regions, with higher SampEn values under Y-D. Similarly, the FuzzyEn results (as shown in Figure 12) showed significant differences in these regions between conditions, with higher FuzzyEn values under Y-D.

## 4 Discussion

This study investigated the effects of visual distraction on sustained attention-related EEG activity under a VR classroom-based CPT paradigm. By collecting behavioral and EEG data from participants during virtual classroom tasks, we analyzed frequency-domain features, time-domain characteristics, and entropy changes in EEG signals. Behavioral results revealed that under visual distraction conditions, participants exhibited significant increases in impulsive responses, omission errors, and commission errors, while no significant differences in reaction times were observed. These findings suggest that visual distractors significantly impair attentional performance. The absence of significant reaction time differences contrasts with some findings in the ADHD literature (Castellanos et al., 2005), where attentional lapses often manifest as response time variability or delay. In our study, the use of a consistent 1,000 ms interstimulus interval may have moderated time pressure, contributing to stable response latencies. This suggests that response times -based measures in VR environments may be less sensitive to distraction effects unless task timing is more dynamic or unpredictable. Analysis of EEG data demonstrated that visual distraction significantly prolonged P300 latencies, with the most pronounced effects observed at the CPz, Pz, and Oz electrode sites. Significant differences in P300 peak amplitudes were also identified at the Fz, FCz, and Oz electrodes. Furthermore, SampEn and FuzzyEn analyses revealed significant increases in entropy values under distraction conditions compared to non-distraction conditions in the frontal (FP1, FP2, F7, F3, Fz, F4, F8, FT7, FC3, FCz, FC4, FT8), central (C3, Cz, C4, CPz), and parietal (P4, Pz) regions. Both SampEn and FuzzyEn metrics showed consistently higher values in these regions during distraction. Our results can be coherently interpreted within the framework of Attentional Resource Theory (Kahneman, 1973; Wickens, 2008), which posits that attention is a finite cognitive resource. In our VR-CPT paradigm, the introduction of visual distractors created a competitive environment where both the primary task and the distractors competed for these limited resources. The behavioral manifestation of this competition was the significant increase in omission and commission errors, indicating performance degradation due to resource depletion. At the neurophysiological level, three key findings collectively reveal the neural dynamics under resource competition: The prolonged P300 latency under distraction aligns with the well-established view that this component reflects the speed of stimulus evaluation, which slows when attentional resources are depleted (Polich, 2007). The observed changes in P300 amplitude at specific electrodes may reflect a dynamic reallocation strategy of these scarce resources (Kok, 2001). Most significantly, the widespread increase in EEG entropy (SampEn and FuzzyEn) provides profound insights into neural adaptive compensation. Rather than indicating system disorganization, elevated entropy suggests that the brain successfully recruited more complex and flexible neural networks to compensate for the attentional drain caused by distractors (McIntosh et al., 2008; Garrett et al., 2013). This enhancement in neural complexity serves as an electrophysiological marker of the brain’s enhanced adaptability and dynamic regulatory capacity—a hallmark of an efficient nervous system striving to maintain task performance amidst competing demands.

By analyzing EEG activity across frequency bands, event-related potential components, and entropy changes in different electrodes and brain regions, we can better understand students’ learning behaviors in virtual environments, thereby providing theoretical foundations for developing new teaching methods and technologies. We observed similar activity distributions in the frontal regions across groups, while differences emerged in the central and parietal regions. Therefore, electrodes Fz, FCz, Cz, CPz, Pz, and Oz were selected as focal points for in-depth analysis. These electrodes cover the prefrontal, central, and parietal regions, which are critical areas associated with attentional processes. In this study, the presence of distractors significantly increased P300 latencies, with the most pronounced effects observed at the CPz, Pz, and Oz electrode sites. This result aligns with findings from Pappalettera et al. (2024), suggesting that visual distraction delays cognitive processing and reduces information processing speed. These findings suggest that the VR-CPT paradigm may be sensitive to transient attentional lapses even in non-clinical populations. The alignment with ADHD-like electrophysiological patterns—particularly in P300 latency—supports its potential utility for evaluating sustained attention in ecologically valid, immersive settings (Castellanos et al., 2005). Numerous studies have consistently reported prolonged P300 latencies in patients with Alzheimer’s disease (AD), mild cognitive impairment (MCI), and ADHD compared to healthy individuals (Tripathi et al., 2015; Parra et al., 2012; Vecchio and Määttä, 2011; Fjell et al., 2007). Furthermore, we measured P300 peak amplitudes at six electrode positions and compared data between N-D and Y-D conditions. Significant differences were observed at the Fz, FCz, and Oz electrodes. This finding contradicts Pappalettera et al.’s (2024), which focused on auditory distraction, potentially indicating modality-specific neural mechanisms that require further investigation. Entropy analyses revealed significant increases in both SampEn and FuzzyEn under distraction conditions compared to non-distraction conditions in the frontal, central, and parietal regions. Elevated entropy values during visual distraction may reflect enhanced neural complexity associated with higher cognitive demands. This observation is consistent with Iinuma et al.’s (2022) findings, where high cognitive load groups exhibited increased entropy in frontal, parietal, and temporal regions, suggesting that distraction in virtual classrooms amplifies neural complexity across multiple brain areas to maintain task focus. Notably, frontal entropy changes align with Wan et al.’s (2021) reports linking frontal EEG dynamical complexity to performance variability during sustained attention tasks. Xu et al.’s (2023) work further supports the association between task difficulty and nonlinear EEG features. Khoshnoud et al.’s (2018) discovery of reduced frontal entropy in ADHD patients clinically corroborates the relationship between entropy and cognitive capacity. Additionally, Tian et al. (2021) demonstrated that irrelevant VR stimuli activate the frontal–parietal-central network, directly mirroring our experimental findings.

Our findings provide theoretical guidance and scientific evidence for developing novel attention assessment and intervention strategies. Particularly in the educational domain, where computer technologies have become increasingly prevalent, students are frequently exposed to visual distractors during learning processes. Understanding the neural mechanisms underlying these distractors is crucial for optimizing learning environments and enhancing academic performance. Future research could further investigate the effects of diverse visual distractor types and individual differences on attentional mechanisms, offering deeper insights for neuroengineering applications in education. For instance, neurofeedback-based EEG tools could be developed to help students monitor and regulate their attentional states in real time. Additionally, classroom layouts and instructional methods may be redesigned to minimize the impact of nonessential visual distractors on student focus.

According to Sendra-Portero et al., virtual classrooms do not significantly differ from traditional classrooms in knowledge acquisition (Sendra-Portero et al., 2013; Browne et al., 2004), nor in post-learning satisfaction (Morice et al., 2020). These findings indicate that virtual classrooms, as an emerging educational tool, are as effective as traditional teaching methods. With ongoing technological advancements, virtual classrooms hold promise for innovation and transformation in education. Our previous studies validated the usability and immersion of virtual classrooms (Wang et al., 2024), showing they can provide a learning experience similar to traditional classrooms while meeting diverse student needs through high interactivity and personalization. Future research should explore how to effectively use visual and other types of distractions in virtual classrooms to promote active learning and deep thinking. In summary, by carefully designing virtual environments and teaching strategies, we can minimize the negative impacts of visual distractions while leveraging their potential benefits to enhance the learning experience and effectiveness. Future studies should continue exploring how to integrate virtual reality technology with educational practices to achieve more efficient and personalized teaching goals.

Our investigation leveraged a multimodal analytical approach—incorporating time-domain (P300), frequency-domain, and nonlinear entropy measures—to delineate the neural mechanisms underlying visual distraction. This comprehensive methodology resonates with a broader paradigm shift in cognitive neuroscience, which emphasizes the integration of advanced computational techniques to decode complex brain functions, as evidenced in recent methodological reviews (Ebrahimzadeh et al., 2022; Ebrahimzadeh and Soltanian-Zadeh, 2024; Ebrahimzadeh and Soltanian-Zadeh, 2025). Specifically, our application of Sample Entropy and Fuzzy Entropy aligns with pioneering work by Ebrahimzadeh et al., among others, who have demonstrated the efficacy of nonlinear dynamics and component-based analysis in capturing neural complexity that conventional linear approaches may overlook (Ebrahimzadeh et al., 2021; Nikravan and Ebrahimzadeh, 2021; Ebrahimzadeh et al., 2023). Whereas sophisticated multimodal frameworks such as simultaneous EEG-fMRI provide exceptional spatial resolution for pinpointing neural sources, particularly in clinical applications like epileptic focus localization (Ebrahimzadeh et al., 2022; Ebrahimzadeh et al., 2019; Ebrahimzadeh et al., 2021; Sadjadi et al., 2021; Sadjadi et al., 2025), our VR-EEG paradigm offers a complementary set of advantages. It prioritizes ecological validity and accessibility, enabling the study of sustained attention within dynamic, real-world-like environments without sacrificing experimental control. The methodological advances captured in these studies—especially the emphasis on high-dimensional feature extraction and machine learning for predictive modeling (Ebrahimzadeh et al., 2023; Ebrahimzadeh et al., 2024)—provide a robust framework for future research. Building on these foundations, subsequent studies could integrate a component-based approach (Ebrahimzadeh et al., 2019; Ebrahimzadeh et al., 2021) via ICA to isolate distraction-specific neural processes prior to entropy quantification, thereby enhancing the interpretability and specificity of the results. Furthermore, the proven efficacy of rTMS in modulating cognitive functions such as attention (Asgarinejad et al., 2024; Ebrahimzadeh et al., 2025) provides a strong rationale for integrating our VR-EEG assessment platform with neuromodulation interventions, potentially creating closed-loop systems for cognitive enhancement. Moreover, the demonstrated capacity of such methods to predict clinical outcomes (Ebrahimzadeh et al., 2023; Ebrahimzadeh et al., 2024) underscores the potential of our VR-EEG platform as a future tool for objective, ecologically valid attention assessment.

While our findings are encouraging, several limitations warrant consideration. First, the small sample size, limited to undergraduate students from Tianjin Medical University, restricts the generalizability of our results to broader populations. Future studies should include participants from diverse backgrounds, such as children or adolescents with ADHD, to enhance sample representativeness and provide more comprehensive insights. Second, given that investigating auditory distractors in virtual reality offers limited advantages for studying sustained attention in isolation, we did not examine the effects of auditory distraction in the VR-CPT paradigm. In real-world classroom environments, auditory distractions such as background chatter, sudden noises, or ambient sounds are prevalent and interact with visual stimuli to influence attention. The lack of auditory components in our design may limit the ecological validity of the task. Future research should incorporate multimodal distractors—including auditory or audiovisual stimuli—to more comprehensively simulate classroom conditions and investigate cross-modal effects on sustained attention. Third, EEG analysis focused on delta, theta, alpha, and beta bands based on their established roles in sustained attention modulation and P300 generation. Gamma oscillations are more susceptible to muscle artifacts in VR setups. Although our study incorporated a counterweight suspension system for the VR headset to reduce pressure on the head, muscle activity can still contaminate the Gamma band. Therefore, current research focuses on four EEG frequency bands. We plan to investigate gamma oscillations using specially designed, high-density EEG systems in future work. Fourth, though ADHD screening (ASRS-v1.1, scores <14) excluded significant symptoms and ensured data were not confounded by undiagnosed deficits, the sample was not stratified by attentional profiles. Future work should incorporate standardized diagnostic interviews to dissociate error patterns (e.g., omissions vs. commissions) across subgroups with varying attentional capacities. Fifth, we did not quantitatively assess participants’ subjective sense of presence or immersion in the virtual classroom. Individual variability in the feeling of “being there” (Ronca et al., 2025) could potentially modulate the impact of distractors and serve as a confounding factor. Future research should incorporate standardized presence questionnaires to evaluate and statistically control for this variable, providing a more nuanced understanding of how subjective experience interacts with distractor effects. Additionally, while we took measures to minimize discomfort (e.g., headset suspension system) and received no reports of severe discomfort, we did not quantitatively assess participants’ subjective experience of comfort or cybersickness when using the combined EEG-VR system. Future studies would benefit from incorporating standardized scales to evaluate and control for these potential confounding factors.

## 5 Conclusion

This study combined VR-CPT and EEG technology to reveal the specific effects of visual distractors on sustained attention and their underlying neural mechanisms. Behavioral results showed that under conditions with visual distractors, the number of impulsive responses, omissions, and extra responses significantly increased, while reaction times did not show significant differences. This indicates that visual distractors exerted a significant negative impact on participants’ attention. Through analysis of the frequency domain characteristics, time domain characteristics, and entropy of EEG data, we found that visual distractors significantly increased the latency of P300, with the most pronounced effects observed at the CPz, Pz, and Oz electrode locations. Analysis of the peak values of the P300 component revealed significant differences at the Fz, FCz, and Oz electrode locations. By analyzing the SampEn and FuzzyEn of EEG data, we found that there were significant differences in SampEn values between distraction and non-distraction conditions in the frontal, central, and parietal regions, with higher SampEn values under distraction conditions. Significant differences were observed between the FuzzyEn values of the frontal, central, and parietal regions with and without distraction, with higher FuzzyEn values under distraction conditions. These findings reveal the specific effects of visual distraction on brain activity, providing valuable insights into the neural mechanisms underlying attention processing and cognitive control. They also offer new ideas and methods for the application of neuroengineering in the field of education, such as how to design more efficient teaching environments and how to reduce distraction during learning. These findings are expected to drive further development and innovation in educational technology.

## Data Availability

The raw data supporting the conclusions of this article will be made available by the authors, without undue reservation.
